# Exploring fascia in myofascial pain syndrome: an integrative model of mechanisms

**DOI:** 10.3389/fpain.2025.1712242

**Published:** 2025-10-27

**Authors:** Vlodeks Gromakovskis

**Affiliations:** ^1^ Pain Medicine Unit, Neurology Department, Association of Health Centers (Veselības Centru Apvienība; VCA), Medical Center Elite, Riga, Latvia; ^2^ Independent Pain Medicine Researcher, Riga, Latvia

**Keywords:** fascia, myofascial pain syndrome, trigger points, chronic pain, connective tissue, biopsychosocial model, central sensitization

## Abstract

Myofascial pain syndrome (MPS) is a leading cause of chronic musculoskeletal pain, yet its mechanisms remain debated. Traditional models emphasized muscle contracture or central sensitization, but growing evidence highlights fascia as a biologically active, pain-relevant tissue. Pathological alterations such as densification, fibrosis, and inflammation may generate nociceptive input and sustain persistent symptoms. To explore this perspective, we conducted a conceptual narrative review of studies published between 2000 and 2025 in PubMed, Embase, Scopus, and Google Scholar. Eligible publications included anatomical, histological, imaging, biomechanical, and clinical investigations, and evidence was synthesized narratively into an integrative model of mechanisms. This mini-review followed the SANRA guidelines for narrative reviews. The literature demonstrates that fascia is richly innervated by nociceptors and sympathetic fibers and undergoes pathological changes in patients with MPS. Imaging and histological studies confirm fibrosis, densification, and inflammatory activity in symptomatic fascia. Mechanistic pathways linking fascia to pain include impaired sliding, abnormal mechanotransduction, and neuroinflammatory sensitization. Clinically, patients exhibit tenderness on fascial palpation, imaging evidence of stiffness, and symptomatic improvement after fascia-focused therapies. These findings suggest that fascia functions as a key peripheral driver in MPS. This concept was first formalized as the ‘integrated hypothesis’ by Simons in 2004. Integrating fascia into existing frameworks reconciles muscle-based and central sensitization models, providing a plausible substrate that initiates nociceptive signaling, perpetuates central adaptations, and interacts with psychosocial influences. This integrative model may explain the heterogeneity of MPS and supports multimodal treatment strategies that combine fascial therapies with central and psychosocial interventions. Although current evidence remains preliminary and heterogeneous, recognizing fascia as a central but interconnected contributor to MPS offers a more comprehensive understanding of this syndrome and a clinically relevant framework for future diagnostic and therapeutic innovation in pain medicine.

## 1 Introduction

Myofascial pain syndrome (MPS) is one of the most common chronic musculoskeletal disorders worldwide, with prevalence estimates ranging from 10% to 20% in the general population and up to 50% in specialized pain clinics (1, 2). Clinically, it is characterized by localized or regional pain, taut bands, and myofascial trigger points (MTrPs) that reproduce referred pain when palpated (3). Although first described in the mid-20th century, MPS remains a controversial entity, with considerable debate regarding its diagnostic criteria, underlying mechanisms, and optimal management (4, 5).

Historically, pathophysiological models of MPS focused primarily on muscle tissue. Early theories attributed symptoms to ischemia, energy crisis, and localized contracture knots caused by dysfunctional motor endplates (6, 7). Electromyographic studies identified spontaneous electrical activity at trigger points, interpreted as abnormal endplate noise (8). These findings supported the view that MPS was primarily a muscle-based disorder. However, treatments targeting muscles directly, such as massage or intramuscular injections, have produced inconsistent outcomes (9).

This early understanding culminated in the formulation of the “integrated hypothesis,” which proposed that persistent endplate dysfunction and localized energy crisis could sustain trigger point activity and pain generation (10).

Later models incorporated central sensitization and psychosocial factors, suggesting that peripheral nociceptive input interacts with the central nervous system to amplify pain (11). Psychological stress, mood disturbances, catastrophizing, and maladaptive coping strategies have been shown to worsen symptom severity and functional impact (12, 13). This biopsychosocial understanding explains some variability in clinical presentations, but does not fully clarify the structural basis of peripheral nociception in MPS.

In the past decade, attention has increasingly shifted toward fascia as a potential primary pain generator (14). Once thought to be merely a passive supportive tissue, fascia is now recognized as a dynamic sensory and mechanometabolic organ (15). Histological and neuroanatomical studies demonstrate that fascia contains dense networks of nociceptors, sympathetic fibers, and mechanoreceptors (16). Moreover, pathologic fascia exhibits fibrosis, densification, altered viscoelasticity, and inflammatory mediator expression-all of which may produce nociceptive input (17, 18).

Clinical and imaging evidence reinforces this perspective. Ultrasound elastography has revealed altered stiffness and reduced sliding between fascial layers in patients with chronic low back pain and neck pain (19). Biopsies of thoracolumbar fascia in chronic pain patients show increased expression of inflammatory cytokines and extracellular matrix remodeling (20). Cadaveric dissections confirm that fascial compartments are richly innervated and capable of transmitting pain signals (21). These findings suggest that fascia is not only involved in force transmission but may itself be a source of pain in MPS.

Fascial dysfunction contributes to pain through multiple potential mechanisms. First, mechanical densification-resulting from impaired hyaluronan metabolism and collagen cross-linking-reduces fascial sliding, leading to stiffness and nociceptor activation (15). Second, fibrosis and thickening increase mechanical stress on embedded sensory nerves (22). Third, neuroinflammatory signaling within fascia amplifies nociceptive drive and may sustain peripheral sensitization (23). Finally, chronic fascial changes may feed into central sensitization, reinforcing pain chronification (24). Thus, fascia provides a unifying peripheral substrate that integrates with muscle and neural processes in MPS.

The therapeutic implications of this shift are significant. Traditional approaches such as trigger point injections (TPI) and dry needling have demonstrated mixed and often modest results in clinical trials (25, 26). However, more recent randomized controlled trials and systematic reviews have reported clinically meaningful pain and disability reductions with dry needling in selected conditions (e.g., neck and low-back myofascial pain), while the mechanistic rationale—including potential fascial contributions—remains debated (27–29). One possible explanation is that these techniques, while effective in some cases, primarily target muscle fibers and may not adequately address pathological fascia. Emerging fascial interventions-including ultrasound-guided hydrorelease (small-volume saline or anesthetic injections into thickened fascia) and hydrodissection (fluid separation of fascial planes, sometimes decompressing nerves)-aim to directly restore fascial mobility and reduce nociceptive signaling (30–32). Although evidence is preliminary, these techniques exemplify a broader conceptual shift toward fascia-focused management of MPS.

Importantly, fascia's role should not be viewed in isolation. Rather, fascia interacts with muscle fibers, peripheral nerves, and central processes in a complex network. This integrative perspective supports a multilevel model of MPS in which fascial changes act as both initiators and perpetuators of pain, synergizing with neural and psychosocial factors (33). Such a model helps explain the heterogeneity of clinical presentations and variable treatment responses observed the purpose of this conceptual review is to explore fascia as a central but interconnected component in the pathophysiology of MPS. By synthesizing anatomical, physiological, and clinical evidence, and by situating fascia within a biopsychosocial framework, we aim to provide an integrative model of mechanisms that may guide both future research and more targeted therapeutic strategies.

## 2 Methods

This mini-review followed the SANRA (Scale for the Assessment of Narrative Review Articles) guidelines for narrative reviews (34). It was conducted as a conceptual narrative review with the aim of integrating current knowledge about fascia into the broader understanding of MPS. The methodology was guided by best practices for literature-based reviews and emphasizes transparency of sources and synthesis.

Search strategy: A comprehensive literature search was performed in PubMed/MEDLINE, Embase, Scopus, and Google Scholar from January 2000 through July 2025. Search terms included “myofascial pain syndrome,” “fascia,” “fascial innervation,” “fascial pathology,” “connective tissue pain,” “biomechanics,” and “chronic musculoskeletal pain.” Additional relevant publications were identified through manual searching of reference lists from key articles and narrative reviews.

Eligibility criteria: Both basic science and clinical studies were considered eligible. This included anatomical, histological, imaging, and biomechanical investigations of fascia, as well as clinical reports and reviews examining fascia in relation to musculoskeletal pain and MPS. Articles exclusively focused on muscle physiology without reference to fascia were excluded. Only peer-reviewed studies published in English were included.

Data synthesis: Findings were synthesized narratively, with a focus on fascia as an innervated, mechanosensitive, and metabolically active structure contributing to MPS. Evidence was integrated into an original conceptual model that situates fascia within a multifactorial biopsychosocial framework of pain. Given the heterogeneity and descriptive nature of the literature, no quantitative pooling of results was attempted.

Ethics and funding: As this article is a literature-based conceptual review, no ethics approval was required. No external funding was received.

## 3 Fascial contributions to myofascial pain syndrome

### 3.1 Anatomy and innervation of fascia

#### 3.1.1 Structural organization and innervation patterns

Fascia forms a continuous three-dimensional matrix surrounding muscles, vessels, and organs, integrating local and global biomechanics (35). Recent meta-analytic evidence demonstrates that connective tissue lesions frequently extend beyond the muscle belly, emphasizing the integral involvement of extramuscular fascia in musculoskeletal injury and repair processes (36). It consists of collagen fibers, elastin, and ground substance rich in hyaluronan, allowing gliding between fascial planes (9).

Fascia is not inert but richly innervated. Thoracolumbar fascia contains free nerve endings, Ruffini corpuscles, and sympathetic fibers (37). Immunohistochemical studies confirm dense nociceptive innervation in both superficial and deep fascial layers (38). Deep fasciae harbor a rich and heterogeneous innervation comprising free nerve endings and low-threshold mechanoreceptors (Ruffini/Pacini), with regional differences across anatomical sites. Histologic evidence indicates increased innervation in pathological fascia. Fascia-resident fibroblasts exhibit mechano-responsiveness mediated by the Yes-associated protein (YAP) and transcriptional coactivator with PDZ-binding motif (TAZ) signaling pathway, which links mechanical strain to fibroblast activation, extracellular matrix remodeling, and transcriptional programs relevant to nociception and tissue stiffness (39, 40).

The abundance of nociceptors explains why fascial palpation reproduces localized and referred pain in MPS (41). This supports fascia as a sensory organ and potential generator of musculoskeletal pain.

## 4 Fascial pathology in myofascial pain syndrome

### 4.1 Fibrosis, densification and inflammatory changes

Pathological fascia demonstrates collagen cross-linking, fiber thickening, and densification, reducing mobility and elasticity (42). These changes impair tissue mechanics and sensitize embedded nociceptors (43). Emerging mechanobiological data suggest that fascial fibrosis, adhesions and impaired layer gliding contribute to nociception. In cadaveric models, fascial hydrorelease reduces gliding resistance between aponeurotic and epimysial layers, offering a plausible mechanism for symptom relief in selected patients (15).

Biopsies of painful fascia reveal upregulation of cytokines (IL-6, TNF-α) and extracellular matrix remodeling enzymes (44). Such biochemical changes perpetuate a cycle of inflammation and pain (45). Recent translational work identified increased expression of YAP/TAZ, TGF-β1 within painful fascia, supporting a chronic inflammatory–fibrotic cascade that alters gliding and nociception (46, 47).

Ultrasound elastography and MRI confirm reduced sliding and increased stiffness of fascial planes in chronic low back and neck pain (13, 48). These findings strengthen the link between structural fascial pathology and clinical symptoms.

## 5 Mechanisms linking fascia and pain

### 5.1 Mechanical mechanisms

Densification of hyaluronan increases the viscosity of the ground substance, impairing gliding between fascial layers and enhancing tissue stiffness (49). Fibrotic remodeling transmits abnormal tension to embedded sensory endings, facilitating peripheral nociceptive drive (50). Mechanotransductive signaling within fascia, mediated by mechanosensitive pathways such as YAP/TAZ and TGF-β1, alters extracellular matrix organization and the local nociceptor milieu, thereby promoting sustained peripheral input that fuels central sensitization (10, 40).

In addition, neurovascular specializations within fascia, including sympathetic fibers and small vessels, provide a structural substrate for persistent nociception and neurogenic inflammation (51–53). Repetitive mechanical loading, impaired sliding, or local ischemia may further upregulate inflammatory gene expression and nociceptor sensitization, linking biomechanical dysfunction to sustained pain (54, 55).

### 5.2 Neuroinflammatory and central mechanisms linking fascia and pain

Inflammatory mediators released within fascial tissues—such as prostaglandins, cytokines, neurotrophins, and growth factors—activate and sensitize nociceptors embedded in the connective tissue matrix, contributing to sustained peripheral sensitization (10, 51, 56). Persistent activation of local fibroblasts and immune cells amplifies cytokine and chemokine release, alters extracellular matrix composition, and increases mechanical stiffness, reinforcing a self-sustaining inflammatory–fibrotic cycle (52, 54).

Mechanotransductive and neuroimmune cross-talk further integrate peripheral and central components of pain processing. Prolonged nociceptive input from fascia may trigger dorsal horn hyperexcitability, alter descending inhibitory control, and induce cortical reorganization of somatosensory maps (53, 57). This cascade underlies central sensitization and the expansion of receptive fields observed in patients with widespread myofascial pain (55, 58, 59).

**Figure 1:**
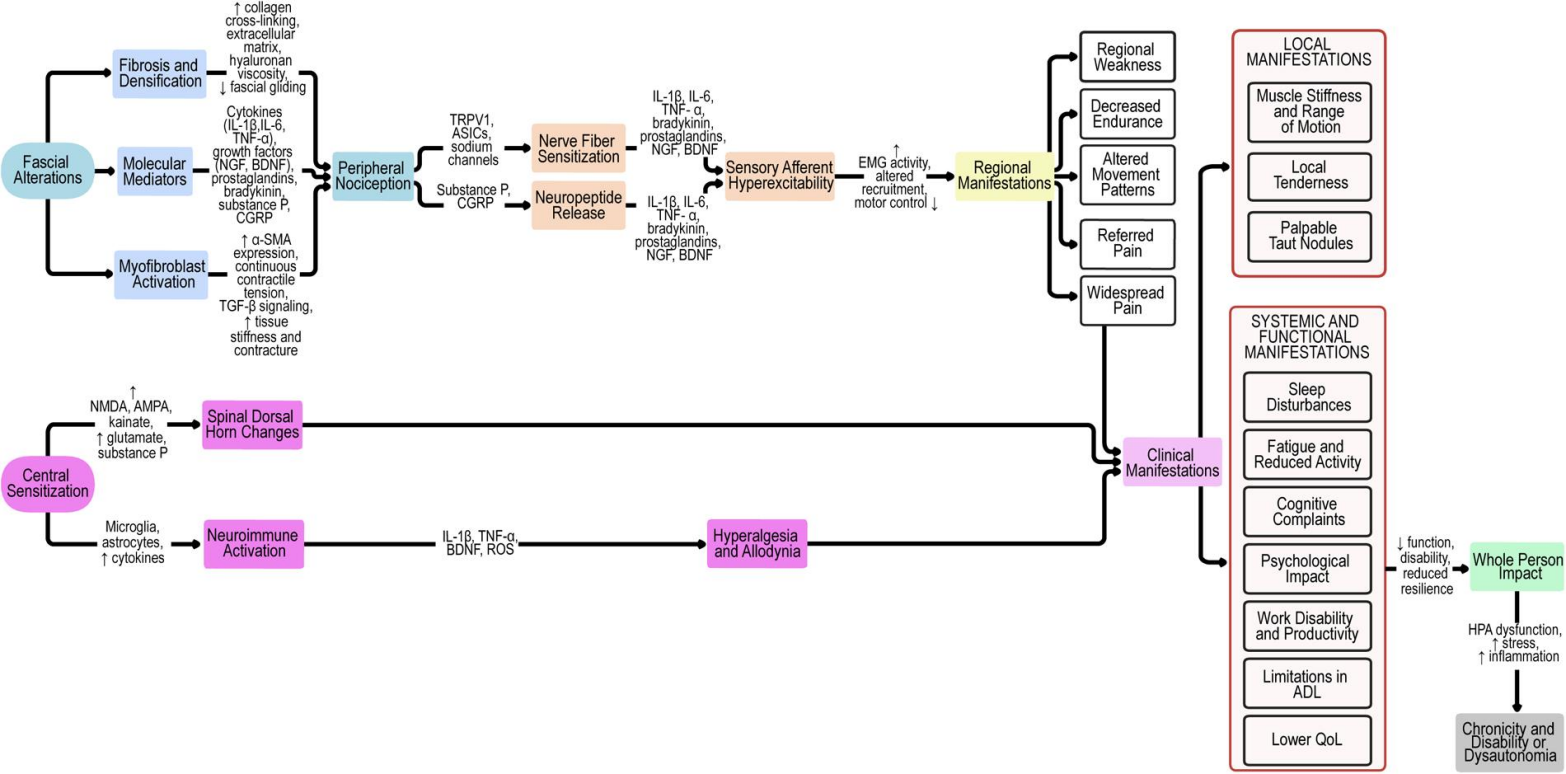
Pathophysiological cascade of fascia-related mechanisms in myofascial pain syndrome. Proposed pathophysiological model linking fascial alterations to peripheral and central sensitization, clinical manifestations, and whole-person impact. Fascial changes (fibrosis, densification, molecular mediators, and myofibroblast activation) drive peripheral nociception and contribute to nerve fiber sensitization, neuropeptide release, and sensory afferent hyperexcitability. These processes result in regional manifestations, local and systemic symptoms, which, together with central sensitization, culminate in widespread pain and whole-person impact. Chronicity and disability arise from maladaptive feedback loops and dysautonomia. ASICs, acid-sensing ion channels; α-SMA, alpha-smooth muscle actin; BDNF, brain-derived neurotrophic factor; CGRP, calcitonin gene-related peptide; EMG, electromyography; HA, hyaluronan; HPA, hypothalamic–pituitary–adrenal axis; IL, interleukin; NGF, nerve growth factor; NMDA, N-methyl-D-aspartate receptor; AMPA, α-amino-3-hydroxy-5-methyl-4-isoxazolepropionic acid receptor; ROS, reactive oxygen species; TGF-β, transforming growth factor beta; TNF-α, tumor necrosis factor alpha; QoL, quality of life; ADL, activities of daily living; TRPV1, transient receptor potential vanilloid 1.

These converging neuroinflammatory and central mechanisms explain how local fascial pathology can evolve into regional or generalized pain and contribute to associated autonomic and affective manifestations. The multiple pathways through which fascia contributes to both peripheral and central sensitization are summarized in the integrative flow diagram (Figure 1).

## 6 Clinical evidence supporting fascia’s role

### 6.1 Palpation and clinical examination

Palpation of densified fascial tissue elicits local tenderness and referred pain patterns, distinct from purely muscular trigger points (60).

Recent ultrasound-based studies have confirmed the correspondence between palpable fascial densifications and sonographic findings, supporting fascia-targeted assessment in clinical practice (59).

### 6.2 Rehabilitation and injection-based approaches

Interventions targeting fascial mobility (e.g., myofascial release, stretching, instrument-assisted soft tissue mobilization) improve pain and function in clinical and pilot trials (61, 62).

Recent randomized and meta-analytic data support fascial-oriented manual therapy as one of the most effective conservative approaches for MPS (63–65).

Beyond manual interventions, ultrasound-guided fascial hydrorelease and hydrodissection techniques have also shown promising biomechanical and clinical outcomes, improving fascial plane gliding and reducing entrapment-related pain (16, 66).

### 6.3 Imaging-guided findings and interventions

Ultrasound and MRI studies show fascial abnormalities improve after manual therapy or injection-based interventions (67). These findings suggest that fascial change, not just muscle treatment, may underlie clinical benefit. Ultrasound (including shear-wave elastography) increasingly documents fascial thickening and reduced sliding in pain phenotypes. Preliminary interventional evidence—ranging from fascial hydrorelease/hydrodissection to fascia-oriented manual approaches—suggests that targeting fascial layers can yield clinically meaningful improvements, although higher-quality trials are required (15). Ultrasound-guided hydrodissection techniques have also shown promising results in neuropathic and myofascial entrapment syndromes, emphasizing the clinical value of fascial plane restoration (66). Modern ultrasonography enables visualization of fascial densifications and altered gliding, bridging palpation with objective assessment (59).

## 7 Toward an integrative conceptual model

Fascial pathology (densification, fibrosis, inflammation) generates nociceptive input that contributes to the peripheral pain source (68).

Beyond a single pathway, MPS may emerge from at least two partially distinct etiopathophysiological routes: a predominantly myogenic route (motor end-plate dysfunction, local metabolic crisis) and a fascial-neurogenic route (innervation-rich fascia, impaired gliding, mechanotransduction). These mechanisms likely co-exist and interact, shaping heterogeneous clinical phenotypes (47, 69).

Persistent peripheral drive facilitates central sensitization, explaining hyperalgesia, referred pain, and chronicity (70).

Psychological and social factors (stress, catastrophizing, inactivity) further amplify pain through neuroimmune and central pathways (71). Fascial dysfunction therefore must be understood as part of an integrative biopsychosocial model (72).

## 8 Discussion

### 8.1 Summary of key findings

The present review highlights fascia as an essential but often overlooked contributor to the pathophysiology of MPS. Integrating recent anatomical, histological, neurophysiological, and clinical research, current evidence supports the view that fascial tissues actively participate in nociception, mechanotransduction, and pain modulation rather than serving merely as passive connective scaffolds (15, 39, 46, 66).

Contrary to classical descriptions of MPS as primarily a muscular disorder, fascial changes such as densification, fibrosis, and altered hyaluronan viscosity are now recognized as key peripheral drivers of pain. These changes impair gliding between fascial layers, elevate local stiffness, and directly stimulate embedded nociceptors (10, 49, 50). Mechanotransductive signaling within fascia—particularly via YAP and TGF-β1—links mechanical strain to fibroblast activation and extracellular-matrix remodeling, reinforcing chronic peripheral input (40, 46).

Fascial inflammation further amplifies nociceptive signaling. Cytokines, prostaglandins, and neurotrophins released from fascia-resident fibroblasts and immune cells sustain a low-grade inflammatory-fibrotic loop that perpetuates peripheral sensitization (51, 52). Prolonged nociceptive drive from fascia to the dorsal horn and higher centers produces neuronal hyperexcitability and reorganization of somatosensory maps, core features of central sensitization (53, 55, 57, 59). Sympathetic and neurovascular networks within fascia may further contribute to sustained nociception and autonomic dysregulation (52, 53).

Clinically, multiple recent randomized controlled trials and meta-analyses support the efficacy of fascial-oriented interventions in restoring mobility and reducing pain. Dry needling, myofascial release, and hydrorelease have demonstrated measurable analgesic and functional benefits in MPS and tension-type headache (27–29). Myofascial and manipulative approaches also show consistent benefit across musculoskeletal pain conditions (64, 65). Diagnostic imaging techniques—including high-resolution ultrasound and elastography—can visualize fascial thickening, densification, and restricted gliding (59, 73).

Recent conceptual and clinical reviews advocate the inclusion of fascia-focused diagnostics and therapies within multimodal pain-management frameworks (47, 69, 74, 75). Collectively, these data establish fascia as a dynamic sensory and biomechanical interface integrating mechanical, neural, and immune pathways of pain. Recognizing its role redefines the etiopathological understanding of MPS and provides a rationale for developing standardized diagnostic and therapeutic protocols that explicitly target the fascial system (15, 47, 59, 65, 66, 69, 75).

### 8.2 Controversies and different schools of thought

Muscle-centric models, while historically important, lack the capacity to explain why some patients with MPS exhibit pronounced fascial stiffness or why palpation of fascial layers reproduces pain (76). Similarly, central sensitization models explain pain amplification but not its initial peripheral drivers. By overlooking fascia, both models risk oversimplification.

In contrast, an integrative model situates fascia as a peripheral initiator of nociceptive input, which interacts with muscle dysfunction and central sensitization. This alignment bridges the gap between local pathology and systemic pain amplification, supporting a biopsychosocial understanding that is clinically relevant (77). The concept echoes other conditions where peripheral tissue changes drive central changes, such as knee osteoarthritis or tendinopathy (78).

Recent work suggests that these models should not be viewed as mutually exclusive but rather complementary, with fascia providing a peripheral driver that interacts with both muscular dysfunction and central sensitization (79).

### 8.3 Added value of a fascia-centered perspective

Recognizing fascia's role has several advantages. First, it clarifies why some interventions traditionally aimed at muscles (e.g., dry needling, TPI) provide inconsistent outcomes-these treatments may not adequately address fascial pathology (80). Second, it validates clinical observations of stiffness, restricted sliding, and pain reproduction upon fascial palpation. Third, it aligns with imaging and histological findings that consistently demonstrate abnormalities in fascia in chronic pain conditions (81, 82). These insights resonate with recent frameworks that position fascia not only as a pain source but as a broader regulatory system interacting with neuroimmune and systemic processes (83).

Furthermore, fascia as a pain source helps explain the heterogeneity of MPS. Some patients present with localized pain and clear MTrPs, while others develop diffuse, persistent pain resistant to conventional therapies. In the integrative model, fascia acts as both a generator of local nociceptive input and a perpetuator of central sensitization (84).

### 8.4 Clinical implications

Clinical implications of fascial dysfunction include the need for improved diagnostic sensitivity and multimodal approaches.

Recent advances highlight the value of ultrasound imaging for visualizing fascial thickness, echotexture, and gliding alterations, which can guide both diagnosis and treatment (59).

Rehabilitation strategies emphasizing fascial mobility—through manual therapy, stretching, and movement retraining—have demonstrated measurable functional gains and pain reduction in controlled trials (63–65).

Moreover, clinician education on the biomechanical and sensory properties of fascia is increasingly recognized as a key element for improving patient outcomes and interdisciplinary management (47, 69).

#### 8.4.1 Diagnosis

Incorporating fascia into diagnostic frameworks means clinicians should not only palpate muscle trigger points but also evaluate fascial layers for stiffness, reduced sliding, or tenderness. Advanced ultrasound techniques such as shear-wave elastography may help identify pathological fascia (85).

#### 8.4.2 Conservative therapies

Manual therapies, stretching, and instrument-assisted fascial release techniques may be particularly effective when fascial densification predominates. Evidence shows improvements in mobility and pain with interventions that specifically target fascia (86, 87).

#### 8.4.3 Interventional approaches

Emerging ultrasound-guided techniques, such as fascial hydrorelease and hydrodissection, aim to restore fascial glide and reduce nociceptive input. While evidence remains preliminary, these procedures embody the shift toward fascia-focused interventions (88, 89).

These insights emphasize that fascia should be considered a therapeutic target in pain medicine, alongside neural and muscular mechanisms (90, 91).

### 8.5 Multimodal integration

Because fascia interacts with neural and psychosocial mechanisms, treatment should be multimodal-combining fascial interventions with exercise, psychological strategies, and central desensitization therapies. This integrative approach aligns with modern pain medicine principles (92). Recent high-level reviews emphasize the need for standardized diagnostic criteria and multimodal treatment strategies integrating fascial assessment (47, 69, 75). Network meta-analyses confirm the efficacy of manipulative and fascial release interventions for MPS, highlighting their role within precision pain management frameworks (65).

### 8.6 Current research gaps

Despite promising insights, the evidence base remains limited and methodologically heterogeneous. Most studies of fascia in MPS are small-scale, observational, or descriptive. Randomized controlled trials investigating fascial interventions are rare, with only isolated examples available (30). Imaging studies demonstrate correlations between fascial stiffness and pain, but causality remains unproven (25). In addition, the absence of validated imaging biomarkers and the lack of longitudinal studies limit the ability to establish causal links between fascial pathology and clinical outcomes (93, 94). Histological studies often rely on small sample sizes and post-mortem tissue, limiting generalizability (95).

Another limitation is the lack of standardized terminology. Terms such as “myofascial pain,” “fasciopathy,” and “fascial dysfunction” are used inconsistently, making it difficult to synthesize findings across studies (96). Moreover, many clinical interventions studied under the label of “myofascial therapy” do not clearly define whether fascia or muscle is the primary target, further complicating interpretation (97). A recent 2025 consensus proposal for a unified definition of the human fascial system underlines the importance of consistent terminology and highlights the urgency of standardization in both research and clinical practice (98).

Finally, psychosocial dimensions of MPS remain underexplored in fascia-focused literature. While fascia provides a compelling peripheral substrate, chronic pain invariably involves central and psychosocial contributions. Ignoring these domains risks replacing one reductionist model with another (99).

### 8.7 Future developments

Future studies are encouraged to address these limitations. Potential research directions include:

1) Standardization of terminology: Clear definitions of fascia-targeted interventions and diagnostic criteria.
2) Mechanistic studies: Using elastography, MRI, and molecular assays to elucidate fascial changes *in vivo*.
3) Large-scale RCTs: Testing fascial therapies against sham or conventional treatments.
4) Multimodal trials: Assessing how fascial interventions integrate with exercise and psychological care.
5) Safety data: Establishing registries for fascial injections and other interventional procedures.

By pursuing these directions, the field can transition from intriguing hypotheses to evidence-based clinical practice (70, 100).

### 8.8 Toward an integrative conceptual model

We propose a model in which fascia is positioned alongside muscle, nerve, and psychosocial factors as a co-equal contributor to MPS. In this framework:

* Fascial pathology (densification, fibrosis, inflammation) generates localized nociceptive input.
* Muscular dysfunction contributes additional peripheral input through contracture and ischemia.
* Central sensitization amplifies pain perception and promotes widespread symptoms.
* Psychosocial influences (stress, catastrophizing, maladaptive coping) sustain and exacerbate pain.

Together, these domains form a multifactorial, integrative model that accounts for both localized trigger point pain and chronic widespread MPS. By situating fascia within this model, clinicians and researchers gain a more comprehensive understanding that may improve diagnostic accuracy, therapeutic targeting, and ultimately patient outcomes (101).

**Figure 2:**
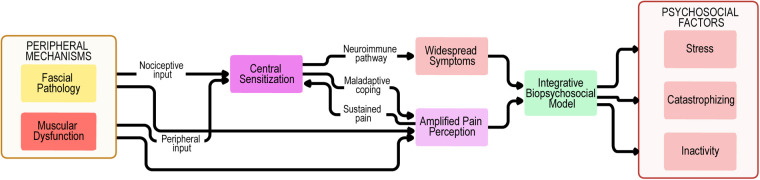
Biopsychosocial model of myofascial pain syndrome. Peripheral mechanisms encompass not only muscular dysfunction and trigger point activity but also fascial pathology, including densification, fibrosis, and impaired sliding properties. These inputs provide nociceptive drive to central sensitization, which is amplified through neuroimmune pathways, maladaptive coping strategies, and sustained pain, leading to widespread symptoms and amplified pain perception. The biopsychosocial model highlights the interplay between fascial, muscular, and central mechanisms, together with psychosocial contributors such as stress, catastrophizing, and inactivity. Feedback loops between these domains perpetuate symptom chronicity, reduced function, and disability in myofascial pain syndrome.

These findings collectively suggest that fascia must be understood as an integral contributor to myofascial pain syndrome. To illustrate these interactions, we propose an integrative conceptual model summarizing peripheral, central, and psychosocial mechanisms (Figure 2).

Emerging technologies such as artificial intelligence and machine learning applied to ultrasound elastography and MRI texture analysis may accelerate biomarker discovery. International initiatives, including those of the International Association for the Study of Pain and the European Pain Federation, could facilitate consensus on terminology and methodological standards (79, 93).

Fascia is not a passive tissue but a biologically active contributor to pain in MPS. Integrating fascia into existing models reconciles historical muscular theories with central sensitization and psychosocial frameworks. While evidence remains preliminary, fascia-centered approaches open promising avenues for diagnosis and therapy. A clear, standardized, and multidisciplinary research agenda is now required to validate and operationalize these concepts.

## 9 Conclusions

MPS continues to challenge clinicians and researchers due to its heterogeneous presentation and lack of universally accepted mechanisms. Traditional models emphasizing muscle contracture and central sensitization provide valuable insights but remain incomplete. This review highlights fascia as a crucial, though often overlooked, component of MPS pathophysiology.

Evidence from anatomical, histological, imaging, and clinical studies demonstrates that fascia is richly innervated, capable of nociceptive signaling, and subject to pathological changes such as densification, fibrosis, and inflammation. These alterations not only generate local pain but also interact with central and psychosocial factors, sustaining chronicity and amplifying symptoms. Integrating fascia into the conceptual framework of MPS offers a more comprehensive model that unites peripheral and central processes within a biopsychosocial context.

Clinically, this perspective suggests that assessment and treatment of MPS should extend beyond muscle fibers to include fascial evaluation and targeted interventions. Manual therapies, rehabilitation strategies, and emerging ultrasound-guided techniques aimed at restoring fascial mobility may provide benefit, particularly when combined with multimodal care addressing central and psychosocial dimensions.

However, the current evidence remains preliminary. Most available studies are small, descriptive, and methodologically heterogeneous, underscoring the need for standardized terminology, rigorous mechanistic research, and adequately powered randomized controlled trials. Only through such efforts can the true role of fascia in MPS be determined and integrated into evidence-based clinical guidelines.

In summary, fascia should be recognized as a central but interconnected contributor to MPS. By embracing an integrative, fascia-informed model, clinicians and researchers may advance understanding, improve patient outcomes, and shape the next generation of therapeutic approaches in chronic musculoskeletal pain.
